# The influence of spatial ability and experience on performance during spaceship rendezvous and docking

**DOI:** 10.3389/fpsyg.2015.00955

**Published:** 2015-07-15

**Authors:** Xiaoping Du, Yijing Zhang, Yu Tian, Weifen Huang, Bin Wu, Jingyu Zhang

**Affiliations:** ^1^China Astronaut Research and Training CenterBeijing, China; ^2^National Key Laboratory of Human Factors EngineeringBeijing, China; ^3^Institute of Psychology, Chinese Academy of SciencesBeijing, China

**Keywords:** spatial ability, experience, task performance, manual rendezvous and docking, astronaut selection, astronaut training

## Abstract

Manual rendezvous and docking (manual RVD) is a challenging space task for astronauts. Previous research showed a correlation between spatial ability and manual RVD skills among participants at early stages of training, but paid less attention to experts. Therefore, this study tried to explore the role of spatial ability in manual RVD skills in two groups of trainees, one relatively inexperienced and the other experienced operators. Additionally, mental rotation has been proven essential in RVD and was tested in this study among 27 male participants, 15 novices, and 12 experts. The participants performed manual RVD tasks in a high fidelity simulator. Results showed that experience moderated the relation between mental rotation ability and manual RVD performance. On one hand, novices with high mental rotation ability tended to perform that RVD task more successfully; on the other hand, experts with high mental rotation ability showed not only no performance advantage in the final stage of the RVD task, but had certain disadvantages in their earlier processes. Both theoretical and practical implications were discussed.

## Introduction

Manned spaceflight continues to receive worldwide attention as it is the primary way for humans to fulfill their curiosity about the universe. Given the risk and cost of space exploration (Steimle and Norberg, 2013), it is widely accepted that astronauts should possess a certain profile of physical and psychological characteristics (Helmreich, 1983; Christensen and Talbot, 1986; Garshnek, 1989; Endo et al., 1994; Rose et al., 1994; Santy and Jones, 1994; Sekiguchi et al., 1994; Ark and Curtis, 1999; Kanas et al., 2002, 2009; Kane et al., 2005; Palinkas, 2007). Among many potential characteristics, spatial cognitive abilities such as mental rotation are generally believed to play a key role in performing space tasks (Shepard and Cooper, 1982; Stransky et al., 2010; Zhang and Cao, 2010). However, questions still remain on when its influence takes place and whether it can be compensated by experience. Answering these questions can expand our knowledge of the inner processes of human cognition as well as improve the current selection and training procedure.

Manual rendezvous and docking (RVD) is crucial for the success of missions such as assembly of large units in orbit, re-supply and exchange of crew of orbital platforms and stations, and lunar/planetary missions (Zhang et al., 2008). Although automation is intensively used in the RVD system, manual RVD remains the most important element to finish space missions and considered as a crucial skill astronauts must master because astronauts are still required to interact with autonomous systems and be ready to take over when automation is failing (Parasuraman and Wickens, 2008; O'Connor and Chief, 2011). In performing a manual RVD task, the operators (astronauts) need to control an active vehicle (the chaser, e.g., the space shuttle) into the vicinity of, and eventually into contact with, a passive vehicle (the target, e.g., the space station). A good manual RVD requires the operator to execute a precise docking (final results) at the lowest fuel cost (the process). As it is a complex tracking and manual control task which also requires special abilities, it is hard to deduce from the theory which form of the ability-performance relationship will occur in performing the manual RVD task. Understanding whether the relationship between spatial ability and performance is transient or enduring has important implications for astronaut selection. If initial differences in performance endure or increase with practice, it may be important to select astronauts based on their spatial ability, whereas if initial relationships between spatial ability and domain-specific performance decrease or vanish after practice, spatial ability may not be emphasized in the selection.

Spatial ability refers to the human cognitive ability to form, retrieve, and manipulate mental models of a visual and spatial nature (Lohman, 1979). It has been found to be an important determinant in a variety of complex manual tasks such as; surgical and medical operations (Eyal and Tendick, 2001; Luursema et al., 2010), driving a car (Lawton, 1994; Gugerty, 1997), piloting an aircraft (Dror et al., 1993; Hunter and Burke, 1994; Wickens and Prevett, 1995), teleoperation tasks such as controlling robot or a robot arm from a remote distance (Lathan and Tracey, 2002; Menchaca-Brandan et al., 2007; Chen, 2010; Long, 2011), and other problem-solving tasks (Oostermeijer et al., 2014). Recently, it has been found that these abilities can enhance the performance of a key component in manual RVD (Tian et al., 2012; Wang et al., 2014). Obviously, spatial abilities are highly essential to perform such a task, as operators need to correctly represent the position of both vehicles in the 3-D space and adjust the position of the active vehicle using a 6-D controller. Studies have found that the mental rotation and perspective-taking ability was significantly correlated with manual RVD performance, and can be used to predict manual RVD performance, indicating that spatial ability is particularly important for accomplishing manual RVD (Wang et al., 2014). However, this study only investigated the final docking results. It is not yet known at what stage of the operation the difference occurs, which indicates the role of spatial ability in the different docking phases and may be useful to the design of display and RVD task as well as individualized training.

Most of the previous studies only examined the effect of spatial ability on relatively inexperienced participants. However, findings based on this population cannot be readily extrapolated to more experienced operators. This is due to a continuous debate on whether the role of cognitive factors such as spatial ability on performance can be compensated by experience (Ackerman, 1987, 1988; Schmidt, 2002; Schmidt and Hunter, 2004). On the one hand, a convergent view has proposed that such abilities can be compensated through experience since more experienced operators will be more reliant on using automatic processes such as chunking and procedure memories in performing these tasks (Shiffrin and Schneider, 1977; Ackerman, 1988), this is particularly the case for tasks of tracking and manual control (Logan, 1989; Hommel, 2000). This argument received empirical support that the predictive power of cognitive abilities diminishes with practice of a task (Fleishman, 1972; Ackerman, 1988). On the other hand, it has been argued that such abilities cannot be compensated, particularly in complex tasks or tasks that require special abilities. It has been found that abilities can be more predictive of performance among experienced workers, especially when the task is more complex (Judge et al., 2010; Zhang et al., 2013) or it requires specific cognitive abilities. For example, studies of tasks with high spatial content such as air traffic control and laparoscopic surgery have found that correlations with spatial ability remain, even after practice (Ackerman, 1992; Ackerman and Cianciolo, 2002; Keehner et al., 2006).

Taken together, the purpose of the present study is to investigate whether spatial ability influences the performance of manual RVD in a similar way among novices and experts. Moreover, we expand upon previous research by investigating the process rather than the final docking results to reveal the inner mechanism of such influence. To guarantee the ecological validity, this study was conducted using a high-fidelity simulator that was actually used for training Chinese astronauts.

## Materials and methods

### Participants

Twenty-seven right-handed male adults (12 novices and 15 experts) with college-level education participated the present study. All 15 novices were postgraduate students with a major in science or engineering (mean age = 23.27, *SD* = 0.96, ranging from 22 to 25). The 12 experts are all aerospace engineers (mean age = 37.25, *SD* = 7.03, ranging from 30 to 48). None of the novices had operated manual RVD task before the experiment. The study was approved by the IRB and all participants signed the informed consent prior to the experiment.

### Measurement of spatial ability

In this study, participants' spatial ability was measured by the Mental Rotation Test (MRT) developed by Vandenberg and Kuse (1978). MRT has been widely used as a reliable measure of the ability to manipulate or transform an image of spatial patterns into other visual arrangements (Vandenberg and Kuse, 1978; Keehner et al., 2006; Luursema et al., 2010, 2012). In each trial, a 3-D target figure and four test figures are shown. Participants need to identify two test figures that are actually rotations of the target figure. In the present study, participants were required to perform 24 trials within 6 min. The score of the test equals is the number of correct responses. The lowest possible score is 0, while 24 is the highest possible score.

### Manual rendezvous and docking task

The manual rendezvous and docking task (RVD) task was conducted on a high fidelity simulator designed for training Chinese astronauts. In performing this task, the participants are required to control the chaser (i.e., Shenzhou spacecraft) to approach the target (i.e., Tiangong space lab) and connect with it.

**Figure 1:**
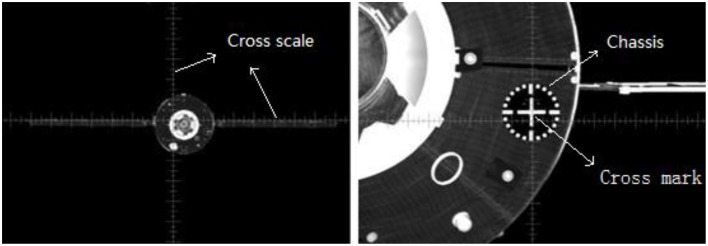
**Cross scale, cross mark, and chassis in the monitor screen**.

The outer view is displayed on an LED monitor and participants can zoom-in or zoom-out by clicking the mouse (the only operation using a mouse). The flight parameters (e.g., speed, relative position, and attitude, etc.) are shown on the periphery of the screen. In the center of the screen, there is a grey cross scale (reticle, Figure 1). When the chaser is close enough to the target, operators can see a white cross marker on the target which contains a central cross and a dial-like chassis. The cross scale and the cross mark are the most important visual cues for operators to estimate the relative position and attitude of the two spacecraft. The two spacecraft are designed in a way that when the white cross mark is aligned in the center of the gray cross scale (within a permissible range), the docking ports on both aircraft can be dovetailed together.

**Figure 2:**
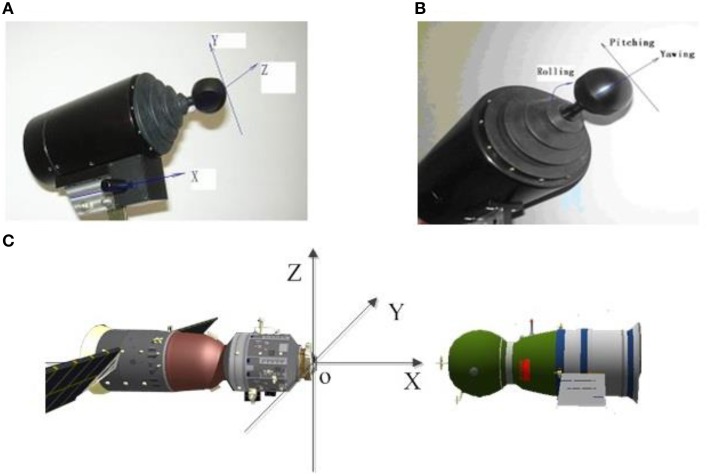
**The handles and coordinate system of rendezvous and docking. (A)** The transitional handle, **(B)** The orientation handle, **(C)** The relevant coordinate system.

Participants can use two handles to control the position and attitude of chaser. The left handle is also called the translational handle and controls the movement, direction and speed of the chaser (see Figure 2A). It is composed of two sticks. Pushing or pulling the lower stick causes the chaser to move forward or backward (along the X axis). Pushing or pulling the upper stick causes the chaser to move up/down (along the Y axis); by moving it toward right or left, the chaser will move toward right or left (along the Z axis). The right handle is also called the orientation handle and controls the attitude of the chaser (Figure 2B). Pulling or pushing it causes the chaser to yaw. By moving it to right or left, the chaser will pitch. In addition, it also controls the rolling of the chaser when operator spins it. The Cartesian orientation was situated in the target, and the relevant coordinate system was provided in Figure 2C.

**Figure 3:**
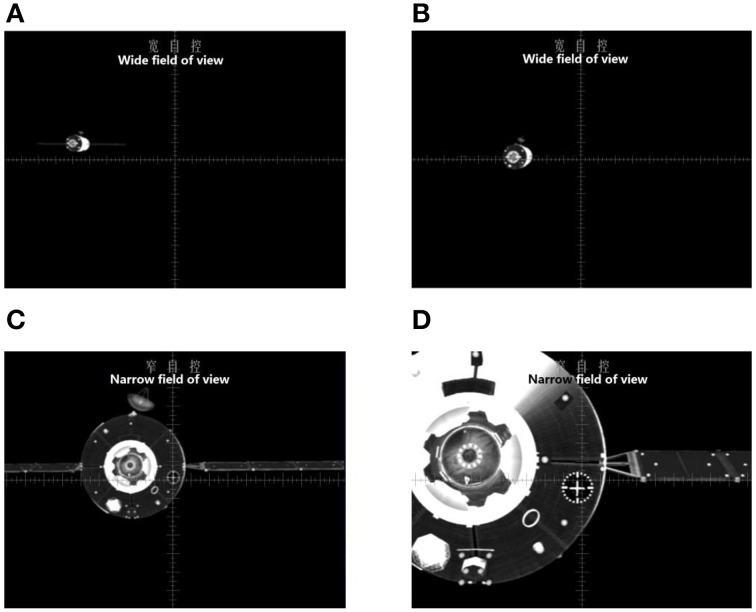
**The image of target spacecraft at different distances. (A)** the distance between the two spacecraft is around 90 m (wide field of view). **(B)** the distance between the two spacecraft is around 60 m (wide field of view). **(C)** the distance between the two spacecraft is around 30 m (narrow field of view). **(D)** the distance between the two spacecraft is around 10 m (narrow field of view).

At the beginning of each scenario, the chaser was 110 m away^1^ from the target (this is generally the point where autopilot terminates and manual control starts). The general operation protocol suggests that the manual RVD task is better performed by following specific objectives in three successive phases. In the first far-distance phase (from 110 m away to 60 m, approximately), the operators should control the chaser until that it faces directly toward the target. In doing so, they should make sure that the image of the target is at the center of the screen. In this phase, they can also start to adjust the altitude of the chaser by referencing some large visual cues of the target (e.g., the shape of the solar panels). In the second moderate distance phase (from 60 m away to 30 m, approximately), the participants can see the cross mark on the target when they zoom-in. In this phase, they can begin to align the cross scale with the cross mark. In doing so, they may need to reduce the approaching velocity (speed along the X-axis) so they can adjust the position and attitude in a controllable manner. In the final approach phase (less than 30 m away), the participants need to adjust the chaser's position and attitude to make the final contact with the target. For a perfect docking, they need to make sure that the cross scale on the monitor is matched perfectly and stably with the cross mark on the target and push the translational handle. Figure 3 shows images of the target spacecraft when the distance between the two spacecraft is around 90, 60, 30, and 10 m, respectively.

In this study, the first performance criterion we used is a composite performance index which is a weighted summed score created by integrating final docking results, angular, and position deviations and fuel consumption. The weighting coefficients had been established by previous studies using expert evaluation and other factor reduction methods (for more details, see Jiang et al., 2011). Ranging from 0 to 1 (a higher score indicates better performance), it is now used for evaluating real training performances. We began by first examining whether spatial ability and experience would influence this overall performance criterion.

Beyond this single and overall performance criterion, we were also interested in exploring the role of spatial ability and experience throughout the whole manual RVD process. To do so, we also investigated three process-related variables including translational deviation (the visual angle between the center of the cross mark and the center of the cross scale^2^), angular deviation (the attitude difference between the two spacecraft), and fuel consumption. Since these variables were too unstable during the very beginning of each scenario (due to engine startup), all these variables were averaged every 10 m from 100 to the 0 m (100–90, 90–80, 80–70……, 10–0, respectively), thus producing 10 process values for each task.

### Procedure

Upon arrival, the participants read and agreed to the informed consent. After they had agreed and signed on the form, they took the MRT test. Next, the novices were trained to perform the manual RVD task. They received oral guidance from a professional RVD trainer to walkthrough the task requirement, control principles, strategies, and performance metrics. Afterwards, they practiced on the simulator to become familiar with the operation. Then they completed three sample scenarios, which were similar to scenarios to be tested in the formal experiment. Expert participants also practiced the same three scenarios to refresh their RVD skill before the formal experiment.

The formal experiment was conducted directly after the practice. Each participant performed an identical set of three scenarios on the manual RVD simulator. The three scenarios differed in the initial relative position and orientation of the chaser and the target. Each scenario lasted for about 7 min and there was a 5-min break between two scenarios. The process variables such as translational deviation, angle deviation, fuel consumption, and final docking results were automatically recorded by the system.

### Data analysis protocol

A two-step process was used to analyze the experiment data. Firstly, we examined the descriptive statistics and zero-order correlations of all variables (process variables were averaged across the whole process). Next, a multiple hierarchical regression analysis was conducted following Aiken and West's (1991) recommended procedure to investigate the influence of spatial ability and experience on overall task performance. In the first step, experience (a dummy coded variable in which −1 represents novice group and 1 represents expert group) and MRT score (centered on grand mean) were entered. In the second step, their interaction term, produced by multiplying the centered MRT score and experience, was entered.

To better understand how spatial ability and experience interacted to influence the operational dynamics, we conducted three multilevel regressions by treating the three process variables (translational deviation, angular deviation, and fuel consumption averaged every 10 m) as dependent variables. The method allows researchers to clearly understand the sources of variances. In the current case, all the process related variables can be predicted by task-level properties (distance) as well as individual-level properties (spatial ability and experience). Rather than downgrading the continuously measured individual difference variable (for example, dividing participants into high- and low- spatial-ability groups to perform a repeated measures ANOVA), multilevel regression can use its original form in the analysis (Raudenbush and Bryk, 2002; Hox, 2010; Zhang et al., 2013).

In the current study, we adopted an exploratory procedure (Raudenbush and Bryk, 2002; Hox, 2010). First, a null model with no predictors at both levels was conducted to quantify the task-level (level 1) and between-individual-level (level 2) components of variance for the three process related variables, respectively. If the results indicate there is a large amount of between individual variances, we proceed to the next step; otherwise, we stop the analysis. Next, a polynomial curve fitting was conducted at level 1 to identify the overall dynamic pattern of all three variables, more specifically, how these variables differed as distance gets closer. In doing so, a level-1 model was constructed where the polynomial terms of distance were entered at level 1. Afterwards, we would further explore whether the intercepts and the shape of these dynamical curves could be influenced by experience, spatial ability and their interaction. In doing so, we entered experience, MRT scores (centered) and their interaction term at level 2 to predict the intercepts and all the coefficients of the level 1 models.

## Results

### Preliminary analysis

Table 1 shows the descriptive statistics and zero-order correlations of all variables. The experience positively correlated with overall performance (*r* = 0.73, *p* < 0.01), and negatively correlated with angular deviation (*r* = −0.56, *p* < 0.01), and fuel consumption (*r* = −0.83, *p* < 0.01), suggesting experts performed significantly better on these criteria. Experience also had a negative correlation with transitional deviation, which did not approach significance (*r* = −0.35, n.s.). However, MRT was not found to have any zero-order correlations with any of the dependent variables nor with experience.

**Table 1:** **Means**, ***SD*****s, and zero-order correlations of all variables**.

	**Mean**	***SD***	**1**	**2**	**3**	**4**	**5**	**6**
Age	29.48	8.46	–					
Experience	−0.11	1.01	0.84^**^	–				
MRT	14.85	3.85	−0.30	−0.32	–			
Overall performance	0.73	0.31	0.63^**^	0.73^**^	0.04	–		
Transitional deviation	0.35	0.83	−0.30	−0.35	−0.12	−0.64^**^	–	
Angular deviation	1.26	1.45	−0.49^**^	−0.56^**^	−0.06	−0.76^**^	0.41^*^	–
Fuel consumption	13.77	5.46	−0.71^**^	−0.83^**^	0.15	−0.63^**^	0.32	0.57^**^

* *p < 0.05*;

** *p < 0.01*.

*Experience is a dummy coded variable in which −1 represents novice group and 1 represents expert group; MRT is the test score of mental rotation task; overall performance was the composite performance index calculated by the mathematical model proposed by Jiang et al. (2011); transitional deviation (YZ deviation) was the distance between the Y and Z axes of the two spacecraft, angular deviation was the angle between the pitch, roll, and yaw axes of the two spacecraft. Overall performance, transitional deviation, angular deviation, and fuel consumption were all averaged over the three scenarios*.

### How experience and spatial ability interact to influence overall task performance

**Figure 4:**
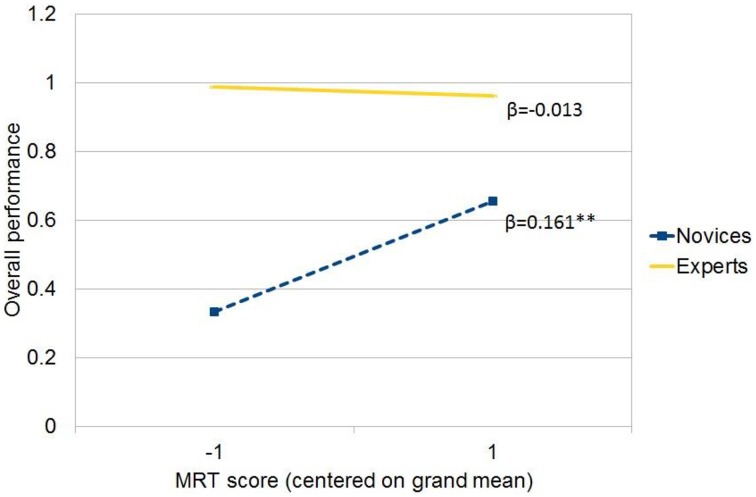
**The joint effect of experience and MRT on overall performance**. (1) ^**^significant at 0.01 level; (2) −1 for Mean MRT score −1 Standard Deviation; 1 for Mean MRT score + 1 Standard Deviation.

We further conducted a hierarchical multiple regression to predict overall task performance. In step 1, the overall model was significant [*R*^2^ = 0.61, *F*_(2, 26)_ = 19.04, *p* < 0.001]. Better overall task performance was related to more experience (β_1_ = 0.83, *p* < 0.001) and higher score of MRT (β_2_ = 0.30, *p* < 0.05). In step 2, adding the interaction term significantly increased the model fit [ΔR^2^ = 0.07, *F*_(1, 23)_ = 5.10, *p* < 0.05] and the interaction term between experience and MRT became a significant predictor (β_3_ = −0.27, *p* < 0.05). We used Aiken and West's (1991) suggested procedure to depict the simple slopes for both groups (see Figure 4 and Table 2). It turned out that the MRT had a positive effect on overall performance for the novice group (β_novice_ = 0.161, *p* < 0.01), but had a non-significant effect for the experts (β_expert_ = −0.013, n.s.).

**Table 2:** **Hierarchical multiple regression predicting overall RVD performance**.

	**Step 1**	**Step 2**
Experience (β_1_)	0.83^***^	0.80^***^
MRT (β_2_)	0.30^*^	0.24
Experience × MRT (β_3_)		−0.27^*^
Δ*R*^2^	0.61^***^	0.07^*^
Total *R*^2^	0.61^***^	0.68^***^

* *p < 0.05*;

*** *p < 0.001*.

*Experience (−1 for novices and 1 for experts)*.

### How experience and spatial ability interact to influence process dynamics

In order to fully understand how the process-related dynamic variables (translational deviance, angular deviance, and fuel consumption) are influenced by the interaction between spatial ability and experience, we conducted a series of multilevel regression analyses. Our first step was to estimate the variance component based on three null models in which no predictors were entered at either levels. According to the index of intra-class correlation, a significant proportion of total variance was between-individuals (transitional deviation = 9.7%, angular deviation = 47.1%, fuel consumption = 39.3%). The existence of such large between-individual variance justified the use of the multilevel analysis.

**Figure 5:**
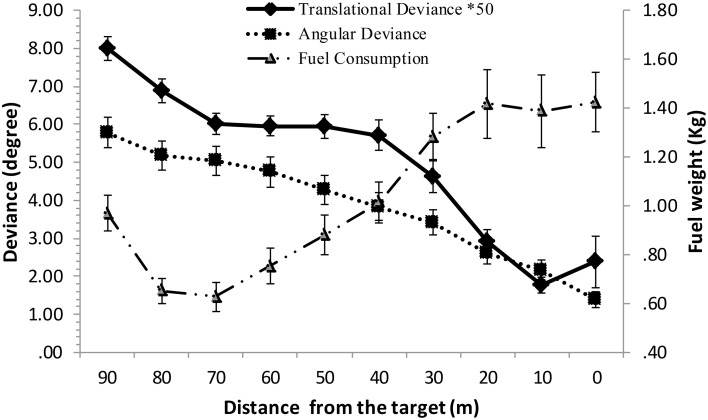
**Overall patterns of three process-related variables**. The value of translational deviance is multiplied by 50 to show it on the same scale with angular deviance.

Next, we attempted to establish a dynamic relationship between distance and all three process-related variables by conducting a polynomial curve fitting analysis. As the operation manual has prescribed a three-phase process, it is reasonable to set the highest order as four (to encompass three stages of change). Further, a closer scrutiny at the distribution of the three variables suggested that a quartic curve might fit the data for translational deviation, a quadric for angular deviation and a cubic curve for fuel consumption (see Figure 5).

Finally, we examined a polynomial model with different ordered terms of distance in an exploratory manner. We started examination of the model including the highest order term:

$$\begin{array}{l} \text{Model }1:Y_{ij}={\text{G}}_{00}+{\text{G}}_{10}\cdot \text{distance}+{\text{G}}_{20}\cdot {\text{distance}}^{2}\\ \;+{\text{G}}_{30}\cdot {\text{distance}}^{3}+{\text{G}}_{40}\cdot {\text{distance}}^{4}+e_{ij}+{\text{u}}_{0} \end{array}$$

In this model, *Y_ij_* is a process-related variable at position i performed by operator j; G_00_ is the intercept; G_10_ ~G_30_ are the regression coefficients of the task-level (level-1) constructs (distance, distance^2^, distance^3^, and distance^4^, distance were all centered before producing the ordered terms). *e_ij_* is the error term at task-level (level 1) while u_0_ is the error term on the between-individual level. If the coefficient of the quartic term (G_40_) is significant, then the test terminates and all items will be maintained for further analyses; if it is not significant, the highest order term will be removed from the model. The test continues to see if the highest order term of the newly truncated model (G_30_ · distance^3^) is significant. This examination will continue until we find a significant highest order term or all polynomial terms of distance is removed.

Based on this analysis, it turned out that a quartic curve well-fit the data for translational deviation, a quadric curve for angular deviation, and a cubic one for fuel consumption. In Table 3, all coefficients are shown in step 1.

**Table 3:** **HLM results predicting process related variables (translational deviation, angular deviation, and fuel consumption)**.

**Parameters**	**Translational deviation**		**Angular deviation**		**Fuel consumption**	
	**Step 1**	**Step 2**	**Step 1**	**Step 2**	**Step 1**	**Step 2**
Intercept (G_00_)	0.12 (0.012)^***^	0.12 (0.011)^***^	4.12 (0.51)^***^	4.22 (0.32)^***^	0.99 (0.11)^***^	0.98 (0.08)^***^
**EFFECT OF LEVEL-1 FACTORS**						
distance (G_10_)	−0.012 (0.003)^***^	−0.011 (0.003)^***^	−0.467 (0.049)^***^	−0.48 (0.043)^***^	0.20 (0.029)^***^	0.21 (0.020)^***^
distance^2^ (G_20_)	−0.006 (0.002)^***^	−0.0064 (0.0017)^***^	−0.026 (0.011)^*^	−0.027 (0.010)^**^	0.010 (0.004)^*^	0.012 (0.004)^**^
distance^3^ (G_30_)	−0.00005 (0.0002)	−0.00005 (0.00023)			−0.007 (0.001)^***^	−0.008 (0.001)^***^
distance^4^ (G_40_)	0.0003 (0.00006)^***^	0.00028 (0.00006)^***^				
**EFFECTS OF LEVEL-2 FACTORS ON SLOPE MEAN**						
Exp (G_01_)		−0.025 (0.011)^*^		−2.01 (0.32)^***^		−0.39 (0.08)^***^
MRT (G_02_)		0.004 (0.003)		−0.009 (0.078)		0.024 (0.02)
Exp × MRT (G_03_)		0.004 (0.003)		0.183 (0.079)^*^		−0.006 (0.02)
**EFFECTS OF LEVEL-2 FACTORS ON SLOPE CHANGE**						
Exp × distance (G_11_)		−0.0041 (0.003)		0.118 (0.043)^*^		−0.083 (0.020)^***^
MRT × distance (G_12_)		0.00027 (0.0006)		0.009 (0.007)		−0.009 (0.004)^*^
Exp × MRT × distance (G_13_)		0.0010 (0.0006)^+^		−0.018 (0.007)^*^		0.013 (0.004)^**^
Exp × distance^2^ (G_21_)		0.0021 (0.0017)		0.023 (0.010)^*^		−0.009 (0.004)^**^
MRT × distance^2^ (G_22_)		−0.00076 (0.0005)		−0.006 (0.003)^*^		−0.002 (0.001)^**^
Exp × MRT × distance^2^ (G_23_)		−0.00074 (0.0005)		0.001 (0.003)		0.003 (0.001)^**^
Exp × distance^3^ (G_31_)		0.00011 (0.00023)				0.001 (0.001)
MRT × distance^3^ (G_32_)		−0.00002 (0.00004)				0.0002 (0.0002)
Exp × MRT × distance^3^ (G_33_)		−0.00001 (0.00004)				−0.0005 (0.0002)^*^
Exp × distance^4^ (G_41_)		−0.00012 (0.00006)^+^				
MRT × distance^4^ (G_42_)		0.00002 (0.00002)				
Exp × MRT × distance^4^ (G_43_)		0.00003 (0.00002)^+^				
PRV	17.7%	25.8%	16.2%	52.4%	9.6%	40.9%
ΔPRV	17.7%	8.1%	16.2%	36.2%	9.6%	31.3%

+ *p < 0.10*;

* *p < 0.05*;

** *p < 0.01*;

*** *p < 0.001*.

By establishing the overall dynamic pattern, we therefore can continue to explore whether these dynamics are influenced by spatial ability, experience, and their interactions. Therefore, based on previous established specific polynomial equations, we added experience (−1 for novices, 1 for experts), MRT score (centered on grand mean), and their interactions (produced by multiplying experience and MRT) to predict the mean and the coefficients of each ordered term of distance. Model 2 includes all predictors. Specifically, based on the results of exploratory analyses of step 1, all predictors (0) + (1) + (2) + (3) + (4) are used to estimate the effect for translational deviance, (0) + (1) + (2) are used to estimate the effect for angular deviance, and (0) + (1) + (2) + (3) are used for fuel consumption. All coefficients are shown in step 2 listed in Table 3.

Model 2:

(0) $$Y_{ij}={\text{G}}_{00}+{\text{G}}_{01}\cdot \text{Exp}+{\text{G}}_{02}\cdot \text{MRT}+{\text{G}}_{03}\cdot \text{Exp}\cdot \text{MRT}+e_{ij}+{\text{u}}_{0}$$

(1) $$+\left({\text{G}}_{10}+{\text{G}}_{11}\cdot \text{Exp}+{\text{G}}_{12}\cdot \text{MRT}+{\text{G}}_{13}\cdot \text{Exp}\cdot \text{MRT}\right)\cdot \text{distance}$$

(2) $$+\left({\text{G}}_{20}+{\text{G}}_{21}\cdot \text{Exp}+{\text{G}}_{22}\cdot \text{MRT}+{\text{G}}_{23}\cdot \text{Exp}\cdot \text{MRT}\right)\cdot {\text{distance}}^{2}$$

(3) $$+\left({\text{G}}_{30}+G_{31}\cdot \text{Exp}+G_{32}\cdot \text{MRT}+{\text{G}}_{33}\cdot \text{Exp}\cdot \text{MRT}\right)\cdot {\text{distance}}^{3}$$

(4) $$+\left({\text{G}}_{40}+{\text{G}}_{41}\cdot \text{Exp}+{\text{G}}_{42}\cdot \text{MRT}+{\text{G}}_{43}\cdot \text{Exp}\cdot \text{MRT}\right)\cdot {\text{distance}}^{4}$$

**Figure 6:**
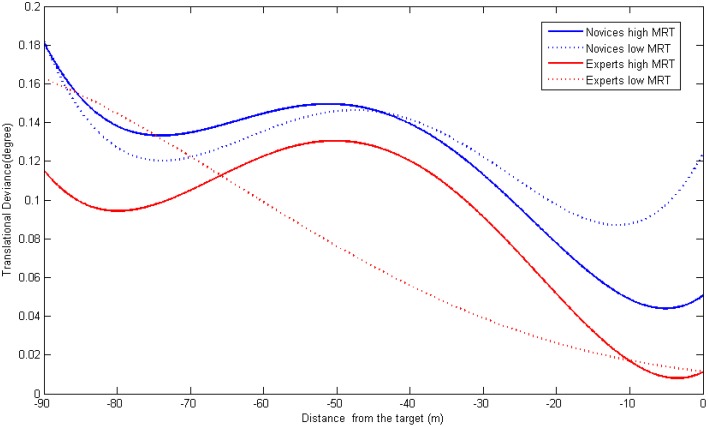
**The joint influence of experience and spatial ability on translational deviation dynamics**.

In predicting translational deviance (see Figure 6), we found a main effect of experience (G_01_ −0.025, *p* < 0.05) in predicting the intercept (Slope Mean) suggesting that experts deviated less throughout the whole process. We also observed that the interaction term was approaching significance to predict the quartic term of distance (G_43_ = 0.00003, *p* = 0.06). By plotting the curves of different groups, we found some divergent patterns. For novices, while there was no big difference in the initial stages, those scoring high on MRT test made less deviance during the end phase as compared to those having a low MRT score. For experts, while there was no difference at the end of the docking process, experts having different levels of spatial abilities showed different temporal dynamics of translational deviance at the early stages.

**Figure 7:**
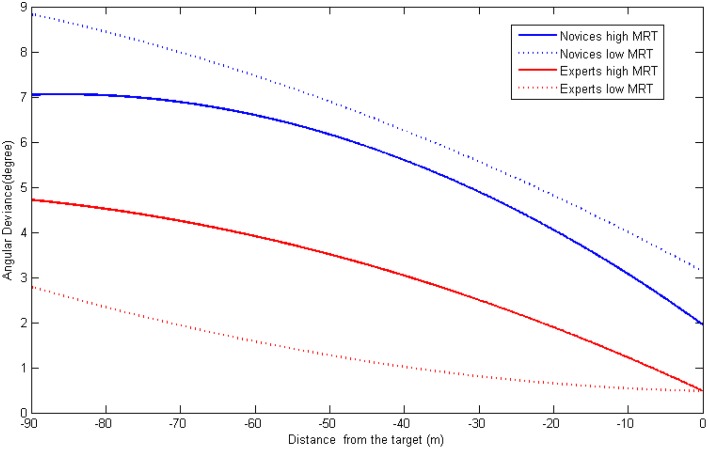
**The joint influence of experience and spatial ability on angular deviation dynamics**.

In predicting angular deviance (see Figure 7), we found experience (G_01_ = −2.01, *p* < 0.001) and the interaction term (G_03_ = 0.183, *p* < 0.05) can predict the intercept (Slope Mean). While experts deviated less throughout the whole process, the effect of spatial ability on experts and novices was the opposite: high MRT novices performed better than their low MRT counterparts, while high MRT experts made more deviances during the process. The interaction term cannot predict the coefficient of the quadric term (G_23_ = 0.001, n.s.), however, it predicted the linear term (G_13_ = −0.018, *p* < 0.05). By plotting the curves of different groups, we found more detailed information regarding this relationship. For novices, participants having high MRT scores had a better rate of accuracy during the whole process constantly. For experts, while high MRT participants did worse in the initial stages, they “caught up” with their low MRT counterparts at the end of the process.

**Figure 8:**
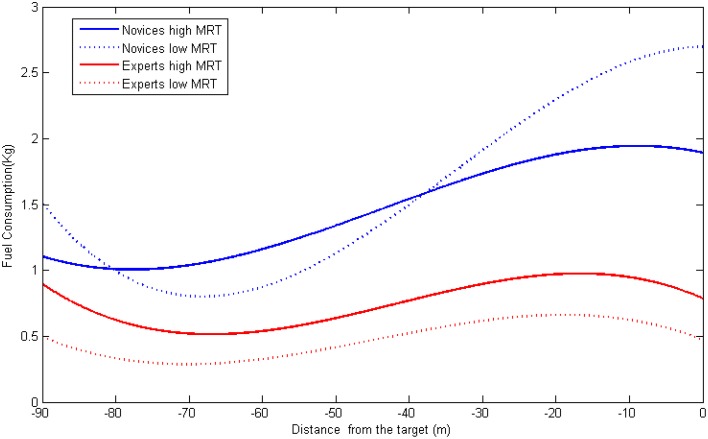
**The joint influence of experience and spatial ability on fuel consumption dynamics**.

In predicting fuel consumption (see Figure 8), the HLM also showed a main effect of experience on the slope mean (G_01_ = −0.39, *p* < 0.001) suggesting experts consumed less fuel during the whole process. In addition, the interaction term of experience and MRT significantly predicted the cubic term of distance (G_33_ = −0.0005, *p* < 0.05). Figure 8 depicts the relationship distance and fuel consumption among novices and experts having different levels of spatial visualization ability. For novices, the difference in fuel consumption in the early stages was small in magnitude between participants having high and low MRT scores; however, when it was approaching the target, those of high MRT scores consumed much less fuel as compared to those of low MRT scores. For experts, those who scored high on the MRT test had higher rates of fuel consumption throughout the whole process.

## Discussion

In the present study, we examined the joint influence of spatial ability and experience on the overall performance of manual RVD task in a high fidelity simulator. Moreover, we explored how this joint influence impacts the process itself. Several findings are worth discussion.

First, we found consistent evidence that experts performed better than novices, in terms of overall performance, accuracy, and fuel utilization throughout the whole process. This finding is consistent with many previous studies in which experience plays a large role in influencing operators' performance.

However, the two groups were influenced differently by their spatial ability. We found mental rotation ability was positively related to the overall performance for novices. Extending upon a previous study in which spatial orientation ability in the sense of perspective taking was particularly important for accomplishing manual RVD (Wang et al., 2014), the present study showed that mental rotation ability was also important for docking spacecraft. This is probably because operators of manual RVD task need to mentally represent the target in 3D space precisely and align it with the chaser. This kind of task requires similar cognitive demand as the mental rotation task. As a result, those scoring high on mental rotation test can have a more accurate mental representation of the relative position and attitude of target, and therefore perform better maneuvers.

While previous studies only investigated the overall performance results, analyzing the detailed processes can help understand such effects in a specific detailed. According to the multilevel regression analyses, we found interesting results that, while high MRT participants made less angular deviation throughout the whole process, their surpass in translational deviation and fuel consumption only appeared during the last phase (from about 30 m away). To note, a significant property in the last phase is that operators can see the cross mark clearly on their screen. Therefore we can postulate that, while maintaining accurate position and attitude both relied on using novices' mental rotation ability, maintaining an accurate position may require clearer visual cues (cross mark). It is not strange that a positional difference occurred at this stage since without a clear visual image, it is hard to make an accurate alignment with the cross mark and the cross scale. The strange thing is that this seemingly 2D task (matching the centers in the mark and the scale) is influenced by operators' 3D mental rotation ability. One explanation is that the ability to perform 2D and 3D mental rotation share some similar component, therefore the MRT measure used in our study can also be used to predict the participants' performance on a 2D alignment task. Another explanation is that, from a limited cognitive resource perspective, the operators were not merely performing a positional adjustment, they also needed to adjust their attitudes at the same time. In this way, those possessing higher processing capacity (with high MRT scores), may have more surplus cognitive ability to perform the dual task and therefore had better performance.

It is also interesting there was a constant difference in angular deviance among novices having different levels of MRT scores. It suggests that participants with high spatial ability can produce a better representation of the target using very vague visual cues (e.g., solar panel, camera, and the body of the target spacecraft). Moreover, this difference cannot be attenuated or increased by having a clearer vision. The underlining mechanism may deserve further exploration. It is possible that the shape of the current target (a space lab) from the angles that were examined in the present study already offers a good visual cue for mental rotation even when it is seen from a faraway distance. The question still remains whether changing the angles or outlook would improve or hinder. Understanding such an issue can provide important theoretical, as well as practical, contributions.

The overall performance of experts was not found to be influenced by their spatial ability, even experienced operators with low spatial ability could achieve acceptable final performance. This result is consistent with the findings of skill acquisition researchers (Fleishman, 1972; Ackerman, 1988), who have shown that cognitive abilities such as spatial ability are important during the initial phase of learning a new skill, but less important in later phases in which skills become increasingly proceduralized (Shiffrin and Schneider, 1977). This is quite different from other studies in which the relationship between cognitive ability and real world performance (supervisor rating, income, job prestige, etc.) showed a divergent pattern (e.g., Judge et al., 2010; Zhang et al., 2013), namely, the effect of cognitive ability on performance became larger as more experience was gained. One possible explanation is that the task we used in our study is more or less predictable for the experts. Indeed, the effect of cosmic turbulence was simulated in the task, so the movement of the chaser and the target were subjected to certain unpredictable physical influences. However, this level of uncertainty may not be able to influence the final performance outcomes for experts because they may rely on using an automatic process, which requires less mental effort to adjust the position and attitude between the two spacecraft. In this regard, their dependence on using the mental rotation ability can be attenuated.

By analyzing the process related variables using multilevel regression, we can see the situation is more complicated than expected. First of all, if we only look at the right end of the Figures 6–8, we can see the same results revealed by analyzing the overall performance: there is no difference in translational nor in angular deviation at the end point among experts having high spatial ability and experts having low spatial ability. However, if we examine earlier stages, we can find a different pattern: experts with high MRT scores tended to make more angular deviance and consume more fuel from the very beginning and more translational deviation from the mid-point. A possible explanation is that the experts with better spatial abilities have more confidence in controlling the task (or think it is too easy for them to perform) thus they did not devote all their effort in the early stages causing the performance to be undermined. When it comes to the end of the task, they can easily adjust the previously deviated spacecraft to achieve the same level of final docking performance. Further studies are needed to test this explanation by investigating the mental workload (e.g., physiological measures) throughout the entire process. Another explanation is that the mental rotation test used for the evaluation of spatial ability is an object-rotation task, which might be quite similar to the way novices used to perform the RVD task. Therefore, their operation is to adjust the visual display so the target on screen is rotated to the right position. However, the experts might embody the spaceship and perform the RVD task in a manner of self-rotation. In this case, their objective is to rotate their own ship so it is in the correct relative direction to the target. In this situation, they may need to refrain from rotating the object and those who are highly capable of doing that, may suffer from certain disadvantages. Although this explanation is very different from the previous one, it can also be examined by using online workload measures. If the workload of the experts of high MRT is high, it suggests that they are doing irrelevant processing (e.g., inhibits rotating the object mentally). Another way is to add a new spatial ability test in which the self-rotation ability is measured. Moreover, two types of tasks (e.g., controlling a spaceship vs. controlling an object) should be examined so we can uncover whether their performance can be predicted by different spatial abilities.

Nevertheless, the complex effect of mental rotation ability on experts' earlier stage performance suggests that caution must be taken in using such criterion to select astronauts. On the one hand, it is still unknown whether the complex (sometime reversed) effect is from a different way of performing the task (self-rotation vs. object- rotation) or other unmanipulated factors. On the other hand, past studies have suggested that different abilities may be called upon during the process to become an expert. While the importance of specific cognitive abilities may diminish with practice, the role of perceptual and motor abilities has been shown to increase over time (Fleishman, 1972; Ackerman, 1988). Thus a greater effect of motor skills might be expected among experienced operators, relative to those with less experience.

It is also possible that certain aiding tools can be offered to compensate the performance gaps. There has been a lot of research on teaching spatially demanding skills (Ventura et al., 2013; Redick and Webster, 2014), and the results so far are encouraging. For example, Gerson et al. (2001) found that the use of visual aids and computer exercises was successful in improving three-dimensional visualization skills among freshman engineering students. Moreover, bench models and virtual reality are being investigated to use for training laparoscopic skills, and the results have shown transfer to performance in the operating room (Scott et al., 2000; Seymour et al., 2002). Therefore, further research is necessary to illustrate the relationship of individual differences in spatial ability, spatial ability training methods, and manual RVD skill, which can contribute to not only the astronaut selection criterion, but also training of spatial ability to improve space task skills such as manual RVD, robotic arms and so on.

Before engaging in further discussion and conclusion, several limitations of this study must be mentioned. Firstly, although the number of participant (15 novices and 12 experts) is quite large for similar research (e.g., Menchaca-Brandan et al., 2007; Long, 2011), it is relatively small as compared to general individual difference studies which may reduce the statistic power. Therefore, the findings, especially those for experts, are subjected to further scrutiny. However, as the pool of the professional operators is rather small, it is quite difficult to examine large sample size. Future research may try to increase the reliability of the cognitive ability test and the number of task scenarios so as to reduce unwanted random errors. Secondly, although this study utilized a high-fidelity simulator in which the interface and the system dynamics were the same as the real RVD task in space, certain environmental factors such as microgravity was not incorporated since the apparatus is both expensive and hard to use. Introducing such influence might produce interesting findings as studies have revealed the influence of microgravity on spatial abilities (Matsakis et al., 1993; Young et al., 1993; Merfeld, 1996; Reschke et al., 1998; Oman, 2007).

Taken together, by scrutinizing the overall dynamics, the results showed, on one hand, novices with high mental rotation ability tended to perform that RVD task more successfully; on the other hand, experts with high mental rotation ability showed not only no performance advantage in the final stage of the RVD task, but had certain disadvantages in their earlier processes. This study raised new questions both theoretically and empirically. Further studies are needed to solve this problem.

### Conflict of interest statement

The authors declare that the research was conducted in the absence of any commercial or financial relationships that could be construed as a potential conflict of interest.

## Footnotes

^1^This is the distance between the docking ports. The distance between mass centers was 120 m.

^2^It was calculated by dividing the longitudinal distance and the lateral distance between the two spacecraft.

## References

[B1] Ackerman P. L. Cianciolo A. T. (2002). Ability and task constraint determinants of complex task performance. J. Exp. Psychol. 8, 194. 10.1037/1076-898X.8.3.194 12240931

[B2] Ackerman P. L. (1987). Individual differences in skill learning: an integration of psychometric and information processing perspectives. Psychol. Bull. 102, 3 10.1037/0033-2909.102.1.3

[B3] Ackerman P. L. (1988). Determinants of individual differences during skill acquisition: cognitive abilities and information processing. J. Exp. Psychol. 117, 288. 10.1037/0096-3445.117.3.2881429345

[B4] Ackerman P. L. (1992). Predicting individual differences in complex skill acquisition: dynamics of ability determinants. J. Appl. Psychol. 77, 598. 10.1037/0021-9010.77.5.598 1429345

[B5] Aiken L. S. West S. G. (1991). Multiple Regression: Testing and Interpreting Interactions. Thousand Oaks, CA: Sage.

[B6] Ark S. Curtis K. (1999). Spaceflight and Psychology. Psychological Support for Space Station Missions. Houston, TX: Behavioral Health and Performance Group; NASA Johnson Space Center.

[B7] Chen J. Y. (2010). Effects of operator spatial ability on uav-guided ground navigation, in 5th ACM/IEEE International Conference on Human-Robot Interaction (HRI), (Osaka: IEEE), 139–140.

[B8] Christensen J. M. Talbot J. M. (1986). A review of the psychological aspects of space flight. Aviat. Space Environ. Med. 57, 203–212. 3516133

[B9] Dror I. E. Kosslyn S. M. Waag W. L. (1993). Visual-spatial abilities of pilots. J. Appl. Psychol. 78, 763. 10.1037/0021-9010.78.5.76317760289

[B10] Endo T. Ohbayashi S. Yumikura S. Sekiguchi C. (1994). Astronaut psychiatric selection procedures: a Japanese experience. Aviat. Space Environ. Med. 65, 916–919. 7832733

[B11] Eyal R. Tendick F. (2001). Spatial ability and learning the use of an angled laparoscope in a virtual environment. Stud. Health Technol. Inform. 81, 146–152. 11317729

[B12] Fleishman E. A. (1972). On the relation between abilities, learning, and human performance. Am. Psychol. 27, 1017 10.1037/h0033881

[B13] Garshnek V. (1989). Soviet space flight: the human element. Aviat. Space Environ. Med. 60, 695–705. 2764853

[B14] Gerson H. B. Sorby S. A. Wysocki A. Baartmans B. J. (2001). The development and assessment of multimedia software for improving 3-D spatial visualization skills. Comput. Appl. Eng. Educ. 9, 105–113. 10.1002/cae.1012

[B15] Gugerty L. J. (1997). Situation awareness during driving: explicit and implicit knowledge in dynamic spatial memory. J. Exp. Psychol. 3:42 10.1037/1076-898X.3.1.42

[B16] Helmreich R. L. (1983). Applying psychology in outer space: unfilled promises revisited. Am. Psychol. 38:445 10.1037/0003-066X.38.4.445

[B17] Hommel B. (2000). The prepared reflex: automaticity and control in stimulus-response translation. Control Cogn. Process. 11, 247.

[B18] Hox J. (2010). Multilevel Analysis: Techniques and Applications. New York, NY: Routledge.

[B19] Hunter D. R. Burke E. F. (1994). Predicting aircraft pilot-training success: a meta-analysis of published research. Int. J. Aviat. Psychol. 4, 297–313. 10.1207/s15327108ijap0404_1

[B20] Jiang T. Wang C. Tian Z. Xu Y. Wang Z. (2011). Study on synthetic evaluation of human performance in manually controlled spacecraft rendezvous and docking tasks, in Digital Human Modeling, ed Duffy V. G. (Orlando, FL: Springer), 387–393. 10.1007/978-3-642-21799-9_43

[B21] Judge T. A. Klinger R. L. Simon L. S. (2010). Time is on my side: time, general mental ability, human capital, and extrinsic career success. J. Appl. Psychol. 95, 92. 10.1037/a0017594 20085408

[B22] Kanas N. Salnitskiy V. Grund E. M. Gushin V. Weiss D. S. Kozerenko O.. (2002). Lessons learned from Shuttle/Mir: psychosocial countermeasures. Aviat. Space Environ. Med. 73, 607–611. 12056680

[B23] Kanas N. Sandal G. Boyd J. E. Gushin V. I. Manzey D. North R. (2009). Psychology and culture during long-duration space missions. Acta Astronaut. 64, 659–677. 10.1016/j.actaastro.2008.12.005

[B24] Kane R. L. Short P. Sipes W. Flynn C. F. (2005). Development and validation of the spaceflight cognitive assessment tool for windows (WinSCAT). Aviat. Space Environ. Med. 76, B183–B191. 15943211

[B25] Keehner M. Lippa Y. Montello D. R. Tendick F. Hegarty M. (2006). Learning a spatial skill for surgery: How the contributions of abilities change with practice. Appl. Cogn. Psychol. 20, 487–503. 10.1002/acp.1198

[B26] Lathan C. E. Tracey M. (2002). The effects of operator spatial perception and sensory feedback on human-robot teleoperation performance. Presence 11, 368–377. 10.1162/105474602760204282

[B27] Lawton C. A. (1994). Gender differences in way-finding strategies: relationship to spatial ability and spatial anxiety. Sex Roles 30, 765–779. 10.1007/BF01544230

[B28] Logan G. D. (1989). Automaticity and cognitive control. Unintended Thought 52–74.

[B29] Lohman D. F. (1979). Spatial ABILITY: A Review and Reanalysis of the Correlational Literature. Stanford, CA: DTIC Document.

[B30] Long L. (2011). Visual spatial abilities in uninhabited ground vehicle task performance during teleoperation and direct line of sight. Presence 20, 466–479. 10.1162/PRES_a_00066

[B31] Luursema J.-M. Buzink S. N. Verwey W. B. Jakimowicz J. (2010). Visuo-spatial ability in colonoscopy simulator training. Adv. Health Sci. Educ. 15, 685–694. 10.1007/s10459-010-9230-y 20455079 PMC2995204

[B32] Luursema J.-M. Verwey W. B. Burie R. (2012). Visuospatial ability factors and performance variables in laparoscopic simulator training. Learn. Individ. Differ. 22, 632–638. 10.1016/j.lindif.2012.05.01215531242

[B33] Matsakis Y. Lipshits M. Gurfinkel V. Berthoz A. (1993). Effects of prolonged weightlessness on mental rotation of three-dimensional objects. Exp. Brain Res. 94, 152–162. 10.1007/BF00230478 8335070

[B34] Menchaca-Brandan M. A. Liu A. M. Oman C. M. Natapoff A. (2007). Influence of perspective-taking and mental rotation abilities in space teleoperation, in Proceedings of the ACM/IEEE International Conference on Human-robot Interaction (Washington, DC: ACM), 271–278.

[B35] Merfeld D. (1996). Effect of spaceflight on ability to sense and control. J. Appl. Physiol. 81, 50–57. 8828647 10.1152/jappl.1996.81.1.50

[B36] O'Connor B. Chief S. (2011). Human-Rating Requirements for Space Systems. Washington, DC: Report NASA/NPR.

[B37] Oman C. (2007). Spatial orientation and navigation in microgravity, in Spatial Processing in Navigation, Imagery and Perception, eds Mast F. Jancke L. (New York, NY: Springer), 209–247. 10.1007/978-0-387-71978-8_13

[B38] Oostermeijer M. Boonen A. J. Jolles J. (2014). The relation between children's constructive play activities, spatial ability, and mathematical word problem-solving performance: a mediation analysis in sixth-grade students. Front. Psychol. 5:782. 10.3389/fpsyg.2014.00782 25101038 PMC4102248

[B39] Palinkas L. A. (2007). Psychosocial issues in long-term space flight: overview. Gravit. Space Res. 14, 25–33. 11865866

[B40] Parasuraman R. Wickens C. D. (2008). Humans: Still vital after all these years of automation. Human factors: J. Hum. Factors Ergon. Society 50, 511–520. 10.1518/001872008X312198 18689061

[B41] Raudenbush S. W. Bryk A. S. (2002). Hierarchical Linear Models: Applications and Data Analysis Methods. Thousand Oaks, CA: Sage.

[B42] Redick T. S. Webster S. B. (2014). Videogame interventions and spatial ability interactions. Front. Hum. Neurosci. 8:183. 10.3389/fnhum.2014.00183 24723880 PMC3972455

[B43] Reschke M. F. Bloomberg J. J. Harm D. L. Paloski W. H. Layne C. Mcdonald V. (1998). Posture, locomotion, spatial orientation, and motion sickness as a function of space flight. Brain Res. Rev. 28, 102–117. 10.1016/S0165-0173(98)00031-9 9795167

[B44] Rose R. M. Fogg L. F. Helmreich R. L. Mcfadden T. J. (1994). Psychological predictors of astronaut effectiveness. Aviat. Space Environ. Med. 65, 910–915. 7832732

[B45] Santy P. A. Jones D. R. (1994). An overview of international issues in astronaut psychological selection. Aviat. Space Environ. Med. 65, 900–903. 7832730

[B46] Schmidt F. L. Hunter J. (2004). General mental ability in the world of work: occupational attainment and job performance. J. Pers. Soc. Psychol. 86, 162. 10.1037/0022-3514.86.1.162 14717634

[B47] Schmidt F. L. (2002). The role of general cognitive ability and job performance: why there cannot be a debate. Hum. Perform. 15, 187–210. 10.1080/08959285.2002.9668091

[B48] Scott D. J. Bergen P. C. Rege R. V. Laycock R. Tesfay S. T. Valentine R. J.. (2000). Laparoscopic training on bench models: better and more cost effective than operating room experience? J. Am. Coll. Surg. 191, 272–283. 10.1016/S1072-7515(00)00339-2 10989902

[B49] Sekiguchi C. Umikura S. Sone K. Kume M. (1994). Psychological evaluation of Japanese astronaut applicants. Aviat. Space Environ. Med. 65, 920–924. 7832734

[B50] Seymour N. E. Gallagher A. G. Roman S. A. O'brien M. K. Bansal V. K. Andersen D. K.. (2002). Virtual reality training improves operating room performance: results of a randomized, double-blinded study. Ann. Surg. 236, 458. 10.1097/01.SLA.0000028969.51489.B4 12368674 PMC1422600

[B51] Shepard R. Cooper L. (1982). “Mental Images and their Transformations, 1982.” (Cambridge, MA: MIT Press).

[B52] Shiffrin R. M. Schneider W. (1977). Controlled and automatic human information processing: II. Perceptual learning, automatic attending and a general theory. Psychol. Rev. 84, 127 10.1037/0033-295X.84.2.127

[B53] Steimle H. Norberg C. (2013). Astronaut selection and training, in Human Spaceflight and Exploration, ed Norberg C. (New York, NY: Springer), 255–294. 10.1007/978-3-642-23725-6_7

[B54] Stransky D. Wilcox L. M. Dubrowski A. (2010). Mental rotation: cross-task training and generalization. J. Exp. Psychol. 16, 349. 10.1037/a0021702 21198252

[B55] Tian Y. Chen S. Wang C. Tian Z. Jiang T. (2012). Correlation of mental rotation ability with the performance of manually controlled rendezvous and docking. Hangtian Yixue yu Yixue Gongcheng 25, 397–402.

[B56] Vandenberg S. G. Kuse A. R. (1978). Mental rotations, a group test of three-dimensional spatial visualization. Percept. Mot. Skills 47, 599–604. 10.2466/pms.1978.47.2.599 724398

[B57] Ventura M. Shute V. Wright T. Zhao W. (2013). An investigation of the validity of the virtual spatial navigation assessment. Front. Psychol. 4:852. 10.3389/fpsyg.2013.00852 24379790 PMC3861692

[B58] Wang C. Tian Y. Chen S. Tian Z. Jiang T. Du F. (2014). Predicting performance in manually controlled rendezvous and docking through spatial abilities. Adv. Space Res. 53, 362–369. 10.1016/j.asr.2013.10.031

[B59] Wickens C. D. Prevett T. T. (1995). Exploring the dimensions of egocentricity in aircraft navigation displays. J. Exp. Psychol. 1, 110 10.1037/1076-898X.1.2.110

[B60] Young L. R. Oman C. M. Merfeld D. Watt D. Roy S. Deluca C.. (1993). Spatial orientation and posture during and following weightlessness: human experiments on Spacelab Life Sciences 1. J. Vestib. Res. 3, 231–239. 8275259

[B61] Zhang J. Li Y. Wu C. (2013). The Influence of individual and team cognitive ability on operators' task and safety performance: a multilevel field study in nuclear power plants. PLoS ONE 8:e84528. 10.1371/journal.pone.0084528 24391964 PMC3877292

[B62] Zhang L. Cao C. G. (2010). The effect of image orientation on a dynamic laparoscopic task, in Proceedings of the Human Factors and Ergonomics Society Annual Meeting (San Francisco, CA: SAGE Publications), 774–778.

[B63] Zhang Y. Xu Y. Li Z. Li J. Wu S. (2008). Influence of monitoring method and control complexity on operator performance in manually controlled spacecraft rendezvous and docking. Tsinghua Sci. Technol. 13, 619–624. 10.1016/S1007-0214(08)70099-3

